# mHealth Early Intervention to Reduce Posttraumatic Stress and Alcohol Use After Sexual Assault (THRIVE): Feasibility and Acceptability Results From a Pilot Trial

**DOI:** 10.2196/44400

**Published:** 2023-07-04

**Authors:** Emily R Dworkin, Macey Schallert, Christine M Lee, Debra Kaysen

**Affiliations:** 1 Department of Psychiatry & Behavioral Sciences University of Washington School of Medicine Seattle, WA United States; 2 Department of Psychiatry Stanford University School of Medicine Palo Alto, CA United States

**Keywords:** mobile health, mHealth, mobile apps, prevention, alcohol use, trauma, sexual violence, rape, mobile phone

## Abstract

**Background:**

Sexual assault is associated with increased risk for both posttraumatic stress (PTS) and alcohol misuse. Mobile health interventions have shown promise in addressing PTS and substance use in trauma survivors and might be a promising strategy in extending the reach of early interventions to individuals who have recently experienced trauma.

**Objective:**

This study assesses the feasibility and acceptability of THRIVE, a mobile health early intervention for recent survivors of sexual assault involving a cognitive behavioral app used daily over 21 days with weekly telephone coaching.

**Methods:**

Twenty adult female survivors of past–10-week sexual assault with elevated PTS and alcohol use were randomized to receive the THRIVE intervention as part of a pilot randomized controlled trial. We sought to understand feasibility by examining rates of completion of intervention activities and testing changes in participants’ self-reported knowledge of key intervention concepts from baseline to after the intervention. We assessed acceptability by collecting self-report ratings of satisfaction with the intervention and app usability in a follow-up survey. The coach took notes during coaching calls to track call content and record participant feedback; these notes were qualitatively analyzed to elaborate on the aforementioned domains.

**Results:**

Feasibility was demonstrated by moderate rates of activity completion: all participants at least opened the app, 19 (95%) of the 20 participants completed at least 1 cognitive behavioral exercise, and 16 (80%) of the 20 participants attended all 4 coaching calls. Participants completed cognitive behavioral exercises on an average of 10.40 (SD 6.52) out of 21 days. The coaching call notes documented participant comments that app-generated reminders increased completion rates. Feasibility was also demonstrated by the finding that knowledge changes occurred from baseline to after the intervention; this indicated that THRIVE was successful in conveying key concepts. Acceptability was demonstrated by high participant ratings of THRIVE’s usability; the ratings corresponded to a B+ usability grade. The coaching call notes documented that usability was increased by the coaching calls, the app exercises’ clarity, and the app exercises’ inclusion of suggestions; however, the coaching call notes also documented that some of the participants found aspects of the app exercises to be difficult or confusing. Acceptability was also demonstrated by participant ratings of satisfaction: most of the participants (15/16, 94%) rated the app as either moderately helpful or very helpful. The coaching call notes documented that the cognitive behavioral activity modules were seen as appealing and that the positive impact of the intervention contributed to participants’ satisfaction.

**Conclusions:**

These findings suggest that THRIVE is feasible and acceptable to survivors of recent sexual assault and that further testing of THRIVE is warranted.

**Trial Registration:**

ClinicalTrials.gov NCT03703258; https://clinicaltrials.gov/ct2/show/NCT03703258

## Introduction

### Background

Sexual assault—defined as forced, coerced, or incapacitated sexual contact—is a common form of trauma among women. According to epidemiological data, 27% to 44% of women are sexually assaulted in their lifetime, and 2.2% to 4.7% are sexually assaulted in a given year [1,2].

Sexual assault is associated with increased risk for both posttraumatic stress (PTS) and alcohol misuse [3]. Approximately 1 in 4 survivors meets criteria for past-year PTS, and 1 in 7 meets criteria for past-year alcohol use disorder [4]. These conditions frequently co-occur in survivors [5], which is concerning given evidence that people with comorbid PTS and alcohol use disorder have poorer treatment engagement and outcomes than people with either disorder alone [6-8]. The co-occurrence of these conditions has been attributed to a functional relationship such that alcohol may be used to cope with PTS [9-11], and this avoidance coping might negatively reinforce further maladaptive alcohol use [10,12-14]. Thus, it is important to identify ways to address these interrelated conditions among sexual assault survivors.

Early intervention (ie, intervention in the months after assault to prevent the development of chronic conditions) could be a key strategy for addressing PTS and alcohol misuse after sexual assault [15,16]. After sexual assault, it is considered common and nonpathological for survivors to display elevated trauma-related symptoms [17]. These symptoms resolve naturally for many but not all survivors [18]. Early interventions are meant to be used at the initial emergence of symptoms—before the symptoms become entrenched and potentially less malleable—to reduce risk for longer-term problems. Early interventions have reduced PTS after sexual assault and other types of traumas and have been more effective among individuals with higher baseline symptoms [16,19]. Effective interventions have typically used cognitive behavioral interventions to target risk factors for PTS, such as avoidance coping and cognitive distortions. However, few sexual assault survivors seek mental health treatment related to their assault [20,21] for reasons that include privacy concerns, shame, and the lack of health insurance [22,23].

Mobile health (mHealth) interventions (ie, interventions delivered via a web platform or smartphone app) [24,25] present an opportunity to increase prompt access to early interventions [26]. Smartphone ownership is pervasive, especially among young adults [27]. Indeed, in a national study, approximately one-third of American adults had an mHealth app installed on their device [28]. Although technology-based early interventions have reduced PTS after other forms of trauma [29], none have reduced posttrauma alcohol misuse, and there is currently no evidence-based mHealth early intervention to address both PTS and alcohol misuse after sexual assault.

### This Study

We conducted a pilot randomized clinical trial of THRIVE, a coached mHealth intervention intended to address PTS and alcohol misuse among female participants who have experienced sexual assault within the prior 10 weeks. This paper reports results related to the goal of assessing intervention feasibility and acceptability in the intervention condition. Regarding feasibility, we hypothesized that (H1) completion rates for daily activities would be similar to those of other web-based interventions (ie, 10 days of a 14-day alcohol use and emotion regulation intervention completed by sexual assault survivors in a study by Stappenbeck et al [30]) and that (H2) participants would show significant learning as evidenced by increases in correct responses to knowledge questions from baseline to after the intervention. Regarding acceptability, we hypothesized that (H3) participants would report above-average usability on a standardized measure and that (H4) most of the participants would respond positively on items assessing satisfaction with the intervention.

## Methods

### The THRIVE Intervention

**Figure 1:**
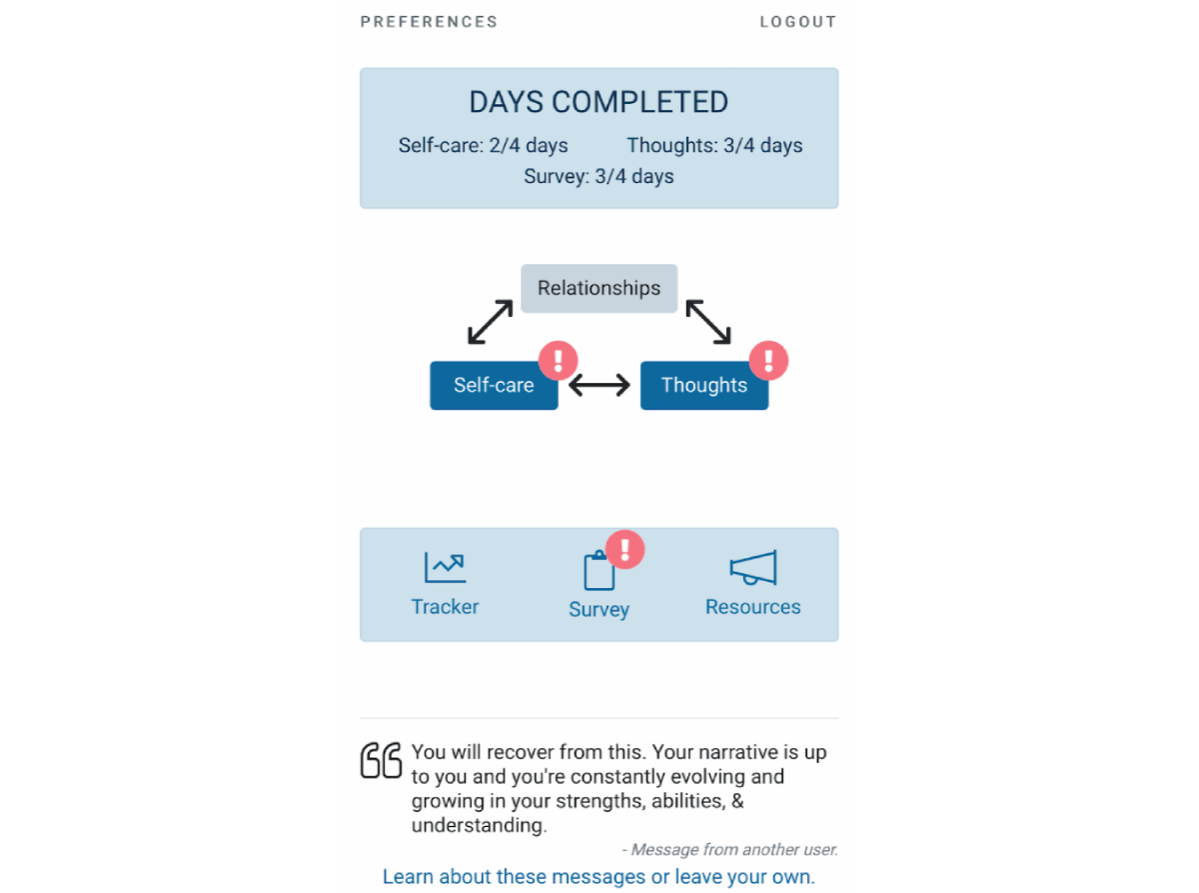
THRIVE app dashboard.

THRIVE is a coached multicomponent mHealth intervention for survivors of recent sexual assault. The app was created by the study team with input from survivors and survivor-serving professionals. The app includes daily exercises with a duration of approximately 5 minutes and 10- to 20-minute weekly coaching calls over a 21-day period, starting within 10 weeks of sexual assault. Total expected user time burden with 100% activity completion and coaching call attendance is 5.4 hours. Figure 1 displays the dashboard of the THRIVE app.

### Intervention Modules

The THRIVE app includes 2 cognitive behavioral modules—cognitive restructuring (*Thoughts*) and activity scheduling (*Self-Care*)—intended to be completed daily and a relationships module (*Relationships*) intended to be completed on an as-needed basis.

#### Cognitive Restructuring Module (Thoughts)

The goal of the cognitive restructuring module is to reduce trauma-related cognitive distortions (eg, self-blame). Cognitive restructuring is a commonly used cognitive behavioral intervention for PTS [31,32] and is a component of relapse prevention for alcohol misuse [33]. On the first day that the cognitive restructuring module is accessed, participants complete a *cognitive restructuring setup exercise*, in which they learn to identify cognitive distortions (consistent with cognitive processing therapy, these were referred to as *stuck points* [34]) using active learning activity and create a list of their cognitive distortions. On subsequent days, users complete a *daily cognitive restructuring exercise*, in which they select one of the cognitive distortions from their list; select and respond to a *challenge question* to restructure the cognition; and identify a new, more adaptive thought based on their challenge question response.

#### Activity Scheduling Module (Self-Care)

The activity scheduling module of THRIVE aims to decrease avoidance behavior (eg, drinking to cope). Activity scheduling is a cognitive behavioral intervention based on reinforcement theories of psychopathology, in which avoidance behavior is negatively reinforcing and reduces opportunities for positive reinforcement [35]. Activity scheduling is a component of effective interventions for both PTS (eg, behavioral activation [36]) and substance misuse [37]. On the first day that the activity scheduling module is accessed, users complete an *activity scheduling setup exercise*: they develop a list of daily activities that are safe, healthy, and important for them to do; complete a brief active learning exercise about the potential risks of drinking to cope; identify activities on their list that might involve heavy drinking and, if desired, append strategies to reduce drinking to those activities; and finally select activities to include on their final activity list. On subsequent days, they complete a *daily activity scheduling exercise*, in which they set a goal to complete ≥1 activities from their list and mark the goal as complete once they engage in that activity.

#### Relationships Module (Relationships)

The THRIVE app includes a relationships module that is intended to be completed on an as-needed basis, rather than daily as with the cognitive behavioral modules. The relationships module includes 5 exercises targeting relational issues that commonly arise after sexual assault: assessing social resources, expressing needs to others, considering the potential risks and benefits of disclosure, coping with negative reactions to disclosure, and identifying strategies to maintain social connections. This module was made optional based on feedback from survivors during the app development process; survivors pointed out that this would allow survivors to engage with these exercises if and when they are needed.

#### Self-Monitoring (Survey and Mood Tracker)

Participants engage in daily self-monitoring of PTS and alcohol use via a brief daily survey. App users can enable SMS text message reminders to remind them to complete the survey at the time of their choice. Users can view a graph of their alcohol use and PTS symptoms plotted together.

#### Supportive Elements

THRIVE includes several elements intended to improve the overall user experience and provide support. First, an *encouraging messages banner*, which displays a rotating set of anonymous encouraging statements from other app users, is continuously visible. Users can submit encouraging messages to be reviewed by staff and displayed. In addition, *resource lists* of therapy and crisis services are available within the app.

#### Coaching Calls

THRIVE offers brief (10-20 minutes) weekly phone support with a coach. Coaching was included in THRIVE because it is thought to increase engagement in mHealth interventions [38], and it has been found to enhance mHealth treatment effects [39-41]. All coaching calls involve a mood check-in, encouragement to complete exercises, discussion and support around completed exercises, and troubleshooting of completion barriers. Safety risk is monitored, and referrals are provided as appropriate; all users receive an offer of referrals in the final call.

### Procedures

The trial of THRIVE was preregistered on ClinicalTrials.gov (NCT03703258). Detailed information about the study procedures is available in the primary outcomes paper [42].

Recruitment started on January 13, 2021, and ended on August 24, 2021. Participants were recruited via mass emails sent to the enrolled student body of the campuses of the University of Washington, fliers posted in community locations, social media advertisements, and referrals from survivor-serving agencies.

Study staff conducted brief phone screenings with 128 individuals to preliminarily assess eligibility criteria. Ineligible participants were offered referrals by the screener. Preliminarily eligible participants (50/128, 39.1%) were guided in installing the study app (but were not able to log in), scheduled for a phone call with a study coach, and sent a consent form link. Participants who provided consent (49/50, 98%) were redirected to the self-report baseline survey to confirm eligibility. Participants who were found to be ineligible (8/49, 16%) were notified via an automated message, paid for survey completion, and offered referrals. Eligible participants (41/49, 84%) were randomized and sent a link to log in to the app. The app automatically displayed the version of the app to which they were randomized: either the full THRIVE app (containing intervention modules as well as self-monitoring and supportive elements) or the control app (containing self-monitoring and supportive elements).

Participants randomized to the intervention condition (20/49, 41%) are the focus of this analysis because they received the hypothesized active elements of the app (ie, the intervention modules). Participants were encouraged to use the app daily over a period of 21 days and attend weekly 10- to 20-minute coaching calls with the first author, a PhD-level licensed clinical psychologist. The 21-day period of use was initiated by logging in to the app for the first time; after 21 days had passed, participants could continue to access all app features other than the self-monitoring elements for 12 weeks but were no longer prompted to access the app.

On day 21 after the baseline assessment, participants were sent the postintervention assessment, which contained the knowledge assessment. Because of a survey programming logic error, participants did not receive the acceptability or usability scales in the postintervention assessment as intended. Participants were recontacted in October 2021 to complete these scales. Of the 20 participants, 16 (80%) completed the scales at this point.

### Ethics Approval and Consent to Participate

The procedures received approval from the University of Washington institutional review board (STUDY00005025). Participants were provided with consent information verbally before screening and in a written information statement before study enrollment. The information provided contained all elements required for informed consent, including a description of the purpose of the research; the voluntary nature of participation, participants’ rights, and the risks of participation; the availability of referral options; data retention and storage information; protections for, and limits, to confidentiality; procedures for reporting complaints and adverse events; and an option to be contacted by the investigators to discuss questions before consenting if needed. Data were deidentified. Participants were paid US $20, US $40, and US $15 in digital gift cards for completing the baseline assessment, postintervention evaluation, and acceptability or usability scales, respectively.

### Participants

The inclusion criteria were as follows: (1) informed consent, (2) self-identification as female, (3) experience of unwanted nonconsensual sexual contact within the past 10 weeks, (4) age ≥18 years, (5) fluency in English, (6) daily smartphone and internet access for 3 weeks and weekly access for 3 months, (7) ≥1 past-month alcoholic drink, (8) ≥1 past–6-month high-risk drinking episode (defined as either >3 drinks on 1 day or >7 drinks in 1 week), and (9) ≥3 symptom clusters endorsed on the PTS Disorder Checklist [43]. The exclusion criteria were active suicidality or psychosis.

### Quantitative Measures

#### Knowledge Change

Knowledge change was assessed at baseline and the postintervention assessment. Two multiple-choice items created for this study assessed knowledge of topics introduced in the cognitive restructuring module: the definition of a *stuck point* (5 response options) and how to cope with an inaccurate thought that leads them to feel negative emotions (4 response options). Two Likert-style items assessed knowledge of topics from the activity scheduling module (ie, the perceived short-term and long-term helpfulness of using alcohol or illicit drugs to cope).

#### Satisfaction

To assess satisfaction, we adapted a scale used in a trial of a similar mHealth intervention (PTSD Coach [44]) by adding items related to our intervention targets and revising existing items to refer to the current app and unwanted sexual experiences. The revised scale included 19 items (eg, “The THRIVE app helped me learn about what I can expect in healing from an unwanted sexual experience”) that were assessed on a 5-point Likert scale ranging from 1 (not at all) to 5 (extremely). The original scale had strong internal consistency with Cronbach α=.96 [44]. Internal consistency in this study was Cronbach α=.95.

#### Usability

The System Usability Scale was used to assess usability [45,46]. This scale consists of 10 items rated on a 5-point Likert scale ranging from 0 (strongly disagree) to 4 (strongly agree). Scores are converted to a range of 0 to 100, and normative letter grades (A+ to F) are available to aid in score interpretation [47]. This scale has demonstrated strong internal consistency (Cronbach α=.91) [45], and internal consistency in this study was Cronbach α=.87.

### Qualitative Data

The first author conducted all weekly coaching calls with participants and took detailed notes during the calls for clinical documentation purposes and to track feedback. As part of these calls, the first author asked participants about their experience of using the app that week. In the final call, the first author asked for more extensive feedback about participants’ overall experience with THRIVE, including aspects they liked and disliked as well as recommendations for changes to THRIVE. These call notes are used as qualitative data in this analysis.

### Quantitative Analysis

#### Completion Rates (H1)

We tested the hypothesis that completion rates for daily activities (ie, cognitive restructuring and activity scheduling) would be similar to those of previous web-based interventions. We tested whether the number of days of completion of these exercises was significantly different from a prior daily web-based intervention for a similar population [30]. As THRIVE’s duration is shorter than that of the prior intervention (21 vs 14 days), we examined both the absolute number of days completed (prior intervention: mean 10, SD 4.8 days completed) and the proportion of days completed (prior intervention: mean 71.43%).

#### Knowledge Change (H2)

We tested the hypothesis that participants would show significant learning as evidenced by increases in correct responses to knowledge questions from the baseline assessment to postintervention assessment via the examination of effect sizes and paired sample *t* tests (2-tailed).

#### Satisfaction (H3)

We tested the hypothesis that most participants would respond positively on items assessing satisfaction with the intervention (H3) by using one-sample *t* tests to determine whether satisfaction ratings were significantly better than *neutral*, defined as average item-level responses significantly above the scale midpoint of 2.

#### Usability (H4)

We tested the hypothesis that participants would report above-average usability by conducting one-sample *t* tests of System Usability Scale scores to determine whether the ratings were significantly different from average usability (ie, a score of 68 out of 100 [48]).

### Qualitative Analysis

We used qualitative data from the coaching calls to contextualize and elaborate on the quantitative results. To analyze these data, we used a grounded theory approach [49]. The first author organized the coaching call feedback into collections reflecting the component of the intervention being described (ie, general app, symptom self-monitoring, activity scheduling setup exercise, daily activity scheduling exercise, cognitive restructuring setup exercise, daily cognitive restructuring exercise, coaching calls, and relationships activities). Next, the first and second authors examined the feedback independently and engaged in an open coding process in which they noted potential themes within each section of the intervention. The first and second authors then met to identify and refine first-order themes (ie, themes reflecting specific pieces of feedback about each section of the intervention) and develop a codebook reflecting these themes. The first and second authors then grouped these themes into second-order themes, which represented patterns in first-order themes across intervention components (eg, factors increasing usability). Subsequently, the first and second authors independently coded the feedback using the codebook and then met to resolve discrepancies and update the codebook as needed.

## Results

### Quantitative Results

#### Overview

The survivors randomized to the intervention condition (n=20) were all adults (mean age 21.45, SD 4.31, years) assigned female sex at birth. Participants identified their race as Asian or Asian American (1/20, 5%), Black (2/20, 10%), White (12/20, 60%), or multiracial (5/20, 25%). One-fifth (4/20, 20%) of the participants identified their ethnicity as Hispanic or Latinx. Most of the participants (18/20, 90%) were students.

#### Feasibility

We examined the feasibility of participants engaging with the intervention by assessing completion rates and the success of the intervention in conveying key concepts.

##### Completion Rates (H1)

**Figure 2:**
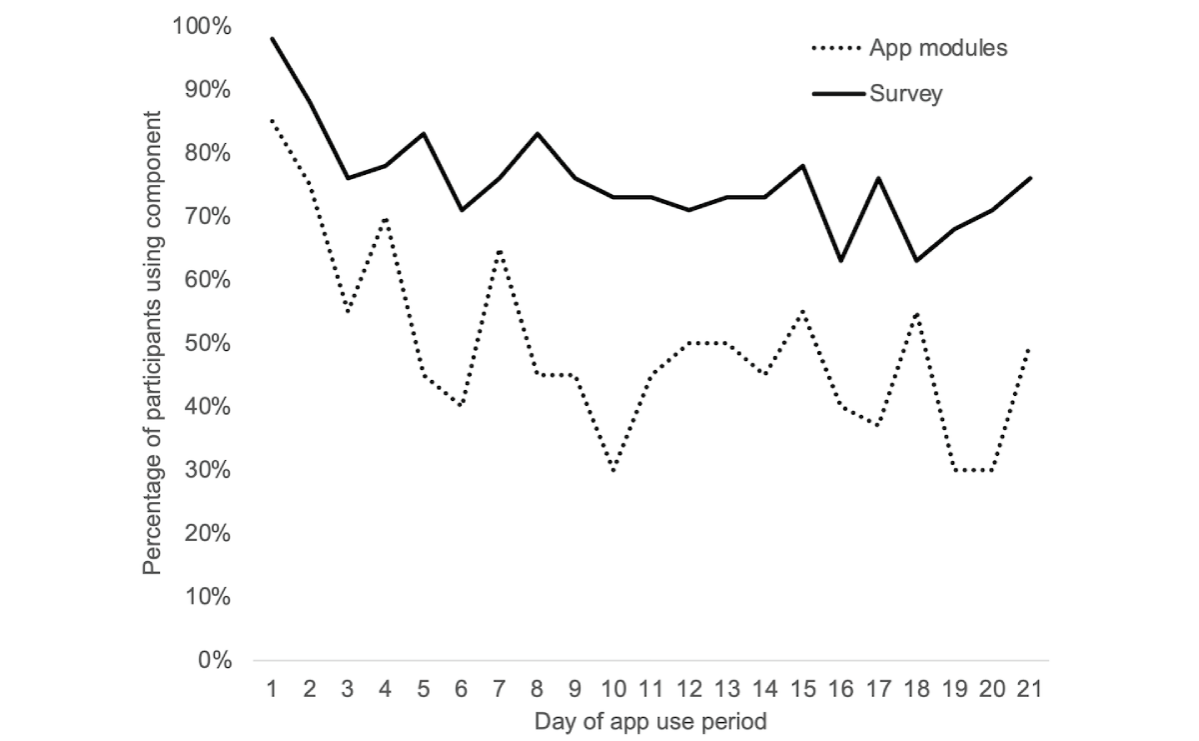
App use rates during the 21-day use period.

Table 1 summarizes completion of specific in-app intervention activities during the 21-day intervention period, and Figure 2 displays changes in use over time.

Of the 20 participants, 19 (95%) completed at least 1 cognitive behavioral exercise in the app, and 3 (15%) continued to complete cognitive behavioral exercises after the intervention period was over. The optional relationships exercises were completed infrequently: of the 20 participants, 8 (40%) completed 1 of 6 exercises, and 1 (5%) completed 2 of 6 exercises. All participants completed at least 2 days of daily self-monitoring surveys in the app and at least 1 coaching call, with 80% (16/20) attending all 4 calls. In support of H1, the average participant-level number of days that at least 1 cognitive behavioral exercise was completed was 10.40 (SD 6.52) days, which was not significantly different than the target number of 10 days of activities completed (t_19_=0.27; *P*=.79). However, against H1, the average participant-level proportion of days completed over the 21-day period was 49.75% (SD 31.36%), which was significantly lower than the 71.43% target completion rate (t_19_=3.09; *P*=.006).

**Table 1:** Intervention exercise completion during the 21-day intervention period (n=20).

Intervention element				Participants completing at least once, n (%)				Number of times completed (multiday exercises only), mean (SD; range)			Number of days completed (multiday exercises only), mean (SD; range)
Guided tour of app (completed 1 time only)				20 (100)				N/A^a^			N/A
**Cognitive restructuring module (completed daily)**				19 (95)				9.55 (7.01; 0-21)			9.20 (6.84; 0-21)
	Setup exercise (completed 1 time only)		19 (95)				N/A			N/A	
	Daily cognitive restructuring exercise		19 (95)				8.55 (6.93; 0-20)			8.20 (6.74; 0-19)	
	Activity scheduling module (completed daily)		19 (95)				32.70 (21.34; 0-65)			9.30 (6.55; 0-19)	
	Setup exercise (completed 1 time only)		19 (95)				N/A			N/A	
	**Daily activity scheduling exercise**		19 (95)				33.45 (21.40; 0-65)			8.60 (6.94; 0-20)	
		Set activity goal			19 (95)	16.75 (10.92; 0-33)			7.75 (5.95; 0-19)		
		Marked activity goal as completed			19 (95)	15.80 (10.55; 0-32)			8.75 (6.50; 0-19)		
**Relationships module** **(completed as needed)**				8 (40)				0.65 (0.81; 0-3)			0.55 (0.60; 0-2)
	Assessing social resources exercise		7 (35)				1.74 (2.58; 0-2)			1.74 (2.58; 0-2)	
	Coping with negative disclosure reactions exercise		1 (5)				0.23 (1.02; 0-1)			0.23 (1.02; 0-1)	
	Weighing costs and benefits of disclosure exercise		2 (10)				0.47 (1.43; 0-1)			0.47 (1.43; 0-1)	
	Maintaining social connections exercise		1 (5)				0.23 (1.02; 0-1)			0.23 (1.02; 0-1)	
	Expressing needs exercise		1 (5)				0.21 (0.93; 0-1)			0.21 (0.93; 0-1)	
Coaching calls (completed weekly)				20 (100)				N/A			3.60 (0.94; 1-4)
Self-monitoring surveys (completed daily; paid)				20 (100)				N/A			14.55 (5.17; 2-21)

^a^N/A: not applicable.

##### Changes in Knowledge (H2)

Descriptive statistics and pre-post comparisons for knowledge items are presented in Table 2.

In support of H2, medium-sized statistically significant changes were observed from the baseline assessment to the postintervention assessment in the knowledge variables, including an increase in the proportion of correct responses to the 2-item cognitive restructuring quiz (*P*=.002) and decreases in perceived short-term and long-term helpfulness of drinking to cope (both *P*=.04).

**Table 2:** Changes in knowledge (N=20).

Variable	Scores			Pre-post comparison			*P* value
	Baseline assessment, mean (SD)	Postintervention assessment, mean (SD)	Within-group Cohen *d*		*t* test (*df*)		
Percentage of correct responses to 2-item cognitive restructuring quiz	0.40 (0.35)	0.82 (0.38)	0.85		3.53 (18)	.002	
Perceived short-term helpfulness of using alcohol to cope^a^	2.60 (1.79)	1.78 (1.17)	−0.53		−2.29 (17)	.04	
Perceived long-term helpfulness of using alcohol to cope^a^	1.80 (1.24)	1.17 (0.51)	−0.61		−2.26 (17)	.04	

^a^Scale ranging from 1=very hurtful to 7=very helpful.

#### Acceptability

##### Usability (H3)

The average usability scale score was 77.66 (SD 16.77) out of 100, corresponding to a usability grade of B+. In support of H4, this score was significantly different from 68 out of 100 (t_15_=2.30; *P*=.04 indicating above-average usability.

##### Satisfaction (H4)

The satisfaction results are summarized in Table 3.

In support of H3, one-sample *t* tests indicated that all item-level average scores were significantly above the scale midpoint of 2.

**Table 3:** Satisfaction survey results (n=16).

Item	Scores (scale range: 1=not at all to 5=extremely)	
	Mean (SD; 95% CI)	Percentage responding “moderately” or higher, n (%)
Helped me learn what I can expect in healing	3.19 (1.05; 2.68-3.70)	11 (69)
Helped me find effective ways of managing my symptoms	3.56 (0.89; 3.12-4.00)	14 (88)
Helped me feel more comfortable in seeking support	3.31 (0.95; 2.84-3.78)	14 (88)
Helped me feel there was something I could do about my healing	3.75 (0.86; 3.33-4.17)	15 (94)
Helped me track my healing (n=15)	3.53 (1.13; 2.98-4.08)	13 (87)
Helped me know when I was doing better or when I was doing worse	3.25 (1.29; 2.62-3.88)	13 (81)
Increased my access to additional resources	3.63 (1.15; 3.07-4.19)	13 (81)
Helped me get more support from people in my life	2.87 (1.31; 2.23-3.51)	10 (63)
Led me to seek counseling or therapy	3.00 (1.03; 2.50-3.50)	12 (75)
Helped me learn new coping skills	3.81 (1.11; 3.27-4.35)	14 (88)
Helped me make positive changes in my life	3.25 (1.00; 2.76-3.74)	13 (81)
Provided practical solutions to the problems I experience	3.44 (0.96; 2.97-3.91)	14 (88)
Helped me overcome the stigma of seeking mental health services	3.56 (1.21; 2.97-4.15)	14 (88)
Helped me better understand what I had been experiencing	3.50 (1.27; 2.88-4.12)	12 (75)
Enhanced my knowledge of healing from an unwanted sexual experience	4.06 (0.93; 3.60-4.52)	15 (94)
Helped me clarify some of the myths about healing from an unwanted sexual experience	3.75 (1.18; 3.17-4.33)	13 (81)
Helped provide a way for me to talk about what I had been experiencing	3.56 (1.15; 3.00-4.12)	12 (75)
Overall, how satisfied were you with the THRIVE app?	3.81 (0.98; 3.33-4.29)	15 (94)
Overall, how helpful was the THRIVE app?	3.69 (0.87; 3.26-4.12)	15 (94)
How helpful were the coaching calls?	3.38 (0.89; 2.94-3.82)	14 (88)

### Qualitative Results

The qualitative analysis of the coaching call notes is summarized in Multimedia Appendix 1. These results elaborate on quantitative findings regarding completion rates, usability, and satisfaction.

#### Completion Rates

The call notes included several factors that participants stated had influenced their completion of activities. In particular, participants noted that the option to set up automated SMS text message reminders and the in-app visual reminders (eg, red exclamation marks placed over incomplete activities) helped them remember to complete activities. Participants also noted specific factors that reduced completion rates for different intervention components. In the daily activity scheduling exercise, some of the participants noted that they did not complete the goals that they had set (and therefore did not mark it as *completed* in the app) owing to outside stresses or responsibilities, because they had simply forgotten, or because they had selected overly ambitious activity goals (because call notes, rather than audio recordings, are analyzed, quotes are presented in the third person, except when the notes included a direct participant quote; revisions were sometimes necessary to enhance clarity and have been denoted by square brackets):

Has been busy, [finds that it is] hard to do schoolwork and also [do] positive activities.

Participant 1, aged 20 years, White

Participants recommended making it possible to set recurring activities and adding more or different types of reminders. In addition, the notes revealed several instances where participants had not accessed the relationships module because they did not notice it in the app:

Didn’t realize that [relationships module] was a clickable section, just discovered it today.

Participant 2, aged 19 years, multiracial

Finally, several of the participants reported during coaching calls that they had been completing exercises outside of the app rather than documenting them in the app:

Listing thoughts in her [iPhone] Notes app about [cognitive distortions] she’s having. She said it’s been really helpful, but it might not show [in the THRIVE app as completed] because she’s not completing the [in-app daily cognitive restructuring] exercises.

Participant 3, aged 18 years, White

#### Usability

Several themes emerged about the usability of THRIVE as discussed in the coaching calls.

##### Aspects of the Intervention That Increased Usability

In general, the coaching call notes reflected positive participant comments about the usability of the app and listed several factors that increased usability. First, the notes documented that several components of the intervention were reported by participants to be especially clear, such as the guided tour of the app on the first day of use and the 2 setup exercises. Second, the notes documented participant comments that the coaching calls helped them make more effective use of the app. The notes specifically documented that participants had said it was useful to talk to the coach to assist them in the process of identifying and challenging cognitive distortions and troubleshooting activity selection and completion:

Talking about breaking down [activity scheduling] tasks into smaller pieces last week really helped.

Participant 4, aged 20 years, White

The coaching call notes documented that participants had said that the app-generated suggestions in the activity scheduling and cognitive restructuring setup exercises were helpful:

Thought having examples/ideas of activities was helpful.

Participant 5, aged 19 years, White

##### Aspects of the Intervention That Decreased Usability and Suggestions

The coaching call notes documented that several of the participants had found various components of the intervention to be confusing, including the activity scheduling setup exercise, the daily activity scheduling exercise, and the daily cognitive restructuring exercise:

Thoughts [exercises] are sometimes it’s not that clear, has to read it over and over. Sometimes the language is not that easily understandable.

Participant 6, aged 23 years, Black

The coaching call notes documented that participants had found several components to be intellectually challenging to complete. In particular, the call notes documented that participants had struggled to think of activities in the activity scheduling setup exercise and had suggested more guidance on setting realistic activity goals:

Would be helpful if it was clearer [in the instructions for selecting activities] that she could do baby steps.

Participant 7, aged 33 years, multiracial and Hispanic or Latinx

The coaching call notes documented that participants had found the daily cognitive restructuring exercise difficult, especially with regard to identifying new cognitive distortions; challenging cognitive distortions; and coming up with new, more balanced thoughts. The notes included recommendations from participants that the app provide more guidance in this exercise:

Sometimes it can be hard to come up with a new thought—start splitting atoms about it.

Participant 7, aged 33 years, multiracial and Hispanic or Latinx

Finally, the coaching call notes documented several bugs or programming issues, such as missing “back” buttons, an inability to set recurring activities in a daily activity scheduling exercise, and issues with the mood tracker lagging or not loading.

#### Satisfaction

The coaching call notes documented high participant satisfaction with THRIVE.

##### Aspects of the Intervention That Made It More Appealing

The coaching call notes documented several aspects of the intervention that participants had found appealing. The notes documented that participants had found the activity scheduling module appealing because of its checklist design and focus on goal setting:

Big fan of checklists. But [it’s a checklist of] things she likes to do.

Participant 8, aged 19 years, Asian or Asian American

The coaching call notes also documented that participants had found the cognitive restructuring module appealing. The notes documented participant comments that they liked the setup part of this module because it was interesting or educational and involved active learning:

Liked [the cognitive restructuring setup exercise], thought it was interesting. Felt like she was learning something, more authentic.

Participant 3, aged 18 years, White

The coaching call notes documented that participants liked the cognitive restructuring process and specifically documented that participants liked the prompts to help them restructure their cognitions as well as the option to choose from multiple prompts:

Feel great just doing the challenge [to her cognitive distortion]—like she’s really overcoming.

Participant 6, aged 23 years, Black

Other aspects specifically identified as appealing in the coaching call notes included the encouraging messages banner, the overall visual design of the app, and the coaching calls.

##### Positive Impact of the Intervention Contributed to Satisfaction

A key factor identified in the coaching call notes as having increased participants’ satisfaction was noticing a broader impact of the app on their recovery. In general, the notes documented that participants described the intervention as a whole as beneficial:

App really helped her get better. Reminded her of who she used to be before things happened, what she liked.

Participant 3, aged 19 years, multiracial

Been amazing...changed my life.

Participant 6, aged 23 years, Black

Coaching call notes for several of the participants identified the activity scheduling module as especially impactful. The notes documented that participants reflected on their coping styles and ultimately engaged in healthy activities because of the module, which led to broader changes in their symptoms:

Kept up with exercising even though things were really busy. Attributes it in part to getting into routine because of the app.

Participant 9, aged 22 years, White

Wasn’t doing activities [before starting to use the app], but doing things now, energy has been boosted.

Participant 6, aged 23 years, Black

[She is now] trying to do stuff she used to do before she got depressed. Taking walks. Trying to be flexible and hold herself accountable.

Participant 7, aged 33 years, American Indian or Alaska Native and Hispanic or Latinx

The coaching call notes also identified that the cognitive restructuring module had been especially impactful and effective in changing participant beliefs:

Starting to feel like it’s not her fault.

Participant 10, aged 23 years, White

Most of [the] stuck points that she had, she doesn’t have anymore, thanks to the app.

Participant 2, aged 19 years, multiracial

Helping her “go to the water, no matter what I end up drinking”—making her more receptive to alternatives.

Participant 7, aged 33 years, multiracial and Hispanic or Latinx

Finally, call notes for several of the participants documented that self-monitoring of symptoms in the daily surveys had been helpful in increasing participant awareness of their emotions and drinking behavior:

Some of the questions got her realizing she drank a lot, [and drank a lot] consecutively, [and led her to realize that] maybe she should cut back.

Participant 11, aged 33 years, Black

##### Aspects of the Intervention That Made It Less Appealing and Suggestions

The coaching call notes for several of the participants documented aspects of the intervention that made it less appealing. Two aspects of the intervention were identified in call notes as emotionally difficult to complete at times: the survey (2 participants’ notes) and the cognitive restructuring module (2 participants’ notes):

Skipped survey for a few days because it was feeling overwhelming to answer questions about who she had conversations with about the assault.

Participant 3, aged 18 years, White

Got overwhelmed by reading through list of stuck points and picking a thought [because she was] in a bad/stressed headspace.

Participant 4, aged 20 years, White

However, it was notable that participants whose call notes reported distress also had call notes describing the app as impactful, despite their distress. In addition, call notes for several of the participants included comments that the cognitive restructuring module was repetitive or tedious and that more variation or a journaling option would make it more engaging:

Said this exercise was tedious, having to write everything out.

Participant 9, aged 22 years, White

Mixing up the activity would make it more helpful. Repetition is only dislike.

Participant 12, aged 18 years, White

Finally, some call notes documented that participants had reported a lack of relevance of aspects of the intervention to their needs.

Said she’s checking things off [on her activity scheduling list] that she’s doing anyway, not that helpful. Said her past therapist helped her with nonavoidance and she’s not really avoiding that much anymore.

Participant 11, aged 33 years, Black

## Discussion

### Overview

The goal of this study was to evaluate the feasibility and acceptability of THRIVE, an mHealth early intervention designed to reduce prospective risk for PTS and alcohol misuse after sexual assault. The results suggest that THRIVE is a feasible intervention that is generally seen positively by participants. These promising results suggest that further development and testing of THRIVE are warranted.

### Feasibility

The study results provide evidence for the feasibility of engaging participants in THRIVE. All intervention condition participants completed at least some cognitive behavioral in-app exercises and attended at least 1 coaching call, with the majority (16/20, 80%) attending all 4 calls. The absolute number of daily activities completed (ie, 10) was similar to that observed in a prior web-based intervention with a similar (but nonacute) sample [30], in support of H1. However, the proportion of days completed was lower because the duration of THRIVE was 21 days compared with the duration of 14 days in the study by Stappenbeck et al [30]. This might indicate that a 14-day intervention period is more realistic than the 21-day period used in THRIVE; future research could explore whether a shorter duration of THRIVE would be effective. It is also possible that participants were less engaged in THRIVE because they were in the acute postassault period, unlike those in the study by Stappenbeck et al [30]. Indeed, in the qualitative results, several of the participants stated that they had outside responsibilities or stresses that took precedence. It is possible that the longer duration of THRIVE allowed participants in the acute phase to receive a similar dose of intervention even with lower frequency of engagement.

In addition, we found that the relationship-focused module was used infrequently. This module was intended to be used on an as-needed rather than daily basis; therefore, participants did not receive regular prompting to access it. The qualitative data revealed that participants did not notice this section owing to its visual design, but those who accessed it found it highly useful. This indicates that the visual design of the app could be improved to make this helpful section more noticeable; periodic prompts could also be added to draw participants’ attention to this module.

We found that THRIVE was successful in conveying its key concepts, providing further evidence of intervention feasibility. Specifically, in support of H2, participants demonstrated increases in comprehension from baseline to the postintervention assessment of the psychoeducational content introduced in the intervention. This indicates that participants were paying attention to the psychoeducational content and that it was presented clearly enough to be retained over the 3-week period. This demonstrates that even a light-touch intervention such as THRIVE can successfully convey important therapeutic concepts.

### Acceptability

Participants generally reported high usability. In the quantitative data, participants gave the THRIVE app a usability score corresponding to a B+ usability grade (in support of H3), highlighting strong usability but room for improvement. This was consistent with the qualitative results: the coaching call notes documented that participants found the exercises to be generally clear and presented in a helpful way but also noted some instances in which specific activities were unclear or difficult. Consistent with prior models of the utility of coaching in mHealth interventions [38], the call notes documented that participants found the coaching calls helpful for clarifying concepts and troubleshooting activities. This feedback provides clear direction for revisions to THRIVE, while also suggesting that coaches may be able to address usability barriers.

Participants also reported high satisfaction with THRIVE. In the quantitative results, most of the participants rated the app (15/16, 94%) and coaching calls (14/16, 88%) as helpful, in support of H4. In the qualitative results, the coaching call notes documented many aspects of the intervention that participants had stated were appealing, including the intervention content and the visual design. Importantly, a key contributor to satisfaction was a perception that the intervention helped with participants’ recovery. It is notable that several of the participants (3/20, 15%) returned to use THRIVE after the intervention period was over, which provides further evidence that THRIVE was seen as useful to participants. Nevertheless, the satisfaction results also indicate areas for improvement, such as identifying ways to make emotionally difficult exercises easier to tolerate and less repetitive. These changes should be explored in future versions of THRIVE.

### Limitations

This study includes several limitations. First, owing to a programming error, participants did not complete acceptability measures immediately after the intervention and, as a result, may have had difficulty remembering their opinion of the THRIVE app. Although most of the participants (16/20, 80%) completed the satisfaction survey when it was resent to them, we were unable to obtain satisfaction data from 4 (20%) of the 20 participants, and it is possible that these participants had lower satisfaction with the intervention. Second, our use of an exclusively female, primarily college-student sample limits our ability to understand feasibility and acceptability among the broader community population that might benefit from using mHealth apps after sexual assault, including men and minoritized racial and ethnic groups. Third, this study was conducted during active COVID-19 restrictions (eg, remote learning for college students and restrictions on social gatherings). Although our ability to enroll survivors of recent sexual assault during this period highlights the feasibility of mHealth interventions for sexual assault survivors even during times of global crisis, the restrictions might have affected app use rates. Fourth, although our mixed methods design is a strength, the use of coaching call notes as qualitative data (rather than separate qualitative interviews) has several biases. In particular, although we obtained feedback from at least 1 coaching call from all participants, the coaching call data do not fully reflect the perspectives of the participants who did not complete all coaching calls (4/20, 20%). As notes were taken by the coach during calls, and the calls were not audio recorded, it is possible that feedback could have been missed or erroneously documented. In addition, because coaching calls were completed by the first author (the trial principal investigator and the creator of the intervention), participants might have felt pressure to report more positive opinions of the intervention. We reduced this likelihood by emphasizing that a study goal was to find ways to improve the intervention; nevertheless, future research should use qualitative interviews conducted by outside evaluators to understand participant opinions.

### Conclusions

These results suggest that THRIVE may be a feasible and acceptable way to deliver an early intervention among sexual assault survivors. If efficacious, THRIVE would be a promising low-barrier strategy to increase the reach of early interventions among this population with high needs.

## Appendix

Multimedia Appendix 1 Summary of qualitative analysis of coaching call notes.

## Abbreviations

mHealth: mobile health
PTS: posttraumatic stress

## Data Availability

The data sets analyzed during this study are not publicly available owing to the pilot nature of the trial but are available from the corresponding author on reasonable request.
