# Supplementary material for: mHealth Early Intervention to Reduce Posttraumatic Stress and Alcohol Use After Sexual Assault (THRIVE): Feasibility and Acceptability Results From a Pilot Trial
Source: JMIR Form Res. 2023 Jul 4;7:e44400. doi: 10.2196/44400 (PMC10354713; doi:10.2196/44400)
Supplement: Multimedia Appendix 1 [file formative_v7i1e44400_app1.docx]

| Theme type | | | Specific themes | |
| --- | --- | --- | --- | --- |
| **Completion rates** | | | | |
|  | Factors that increased completion rates | | - Reminders were helpful - Reminders/notifications in general were helpful - App-generated text reminder was helpful to remember to complete daily activity scheduling exercise | |
|  | Factors that decreased completion rates and suggestions | | - Participant-related factors affected completion of daily activity scheduling exercise - Participant was too busy or stressed - The goals they set were too big, difficult, overwhelming, or unrealistic to complete - Suggestion*:* make it possible to create recurring activities - Forgot to complete the exercise   1. Suggestion*:* add more or different options for receiving reminders - Participant did not notice relationships module - Suggestion: make this section more apparent within the app - Participant completed daily exercises outside of the app | |
| **Usability** | | | | |
|  | Aspects of the intervention that increased usability | | | - Clarity - Setup exercises were clear and concise - App guided tour was simple, straightforward, and clear - Coaching calls helped explain or make better use of the app - Calls were helpful for identifying and challenging stuck points - Calls were helpful for talking through activity selection and completion - Suggestions helped in the setup exercises - Prepopulated activity list in the activity scheduling setup activity was helpful - Liked the suggested list of cognitive distortions that participants could use to build a list of their own distortions to challenge in the cognitive restructuring setup exercises |
|  | Aspects of the intervention that decreased usability and suggestions | | | - Difficulty level of exercises was high - Hard to come up with activities in the activity scheduling setup exercise   1. Suggestion: provide more guidance on setting activity goals - Difficult to identify new cognitive distortions; challenge cognitive distortions; and come up with new, more balanced thoughts in daily cognitive restructuring exercise   1. Suggestion: provide more support in identifying cognitive distortions - Exercises were confusing - Misunderstood the activity scheduling setup exercise or found it confusing - Daily activity scheduling exercise was confusing - Daily cognitive restructuring exercise was confusing/unclear - Difficult to access and understand summaries of exercises - Suggestion: add a user-friendly summary of completed activity scheduling exercises - Bugs and programming issues - Missing “back” buttons - Unable to set recurring activities in daily activity scheduling exercise - Mood tracker lagging or not loading |
| **Satisfaction** | | | | |
|  | | Aspects of the intervention that made it more appealing | - Liked activity scheduling module - Liked that the activity scheduling setup activity involves setting goals - Liked the checklist aspect of the daily activity scheduling exercise - Liked cognitive restructuring module - Found the cognitive restructuring setup activity interesting; liked the educational/active learning focus - Liked going through the process of challenging their thoughts in the daily cognitive restructuring exercise - Liked the questions/prompts in the daily cognitive restructuring exercise and being able to pick from multiple questions/prompts - Liked coaching calls - Liked encouraging messages - Liked visual design | |
|  | | Positive impact of the intervention contributed to satisfaction | - Used skills as suggested by app - More likely to engage in healthy activities because of the daily activity scheduling exercise - The daily activity scheduling exercise helped them perform specific activities that counteracted unhelpful coping style - The relationships module helped them challenge specific stuck points - Began to use coping skills habitually - The daily activity scheduling exercise helped them establish a routine/habit - Led participant to reflect in helpful ways - The daily activity scheduling exercise helped them reflect on how they had previously been coping in positive or negative ways - The cognitive restructuring setup exercise helped them reflect on their way of thinking - The survey helped them reflect on how they were feeling/behaving - Participant experienced a change in symptoms - The intervention as a whole had an impact on their recovery - Changing their behavior via the daily activity scheduling exercise had broader impact on their symptoms - The daily cognitive restructuring exercise changed their way of thinking overall, decreased self-blame, decreased nonblame cognitive distortions, changed their way of coping when cognitive distortions emerge, or changed their emotions - The survey had an impact on their recovery/drinking - The relationships module helped them realize that help would be there if they needed it or helped them appreciate their support system more | |
|  | | Aspects of the intervention that made it less appealing and suggestions | - Emotionally difficult - Emotionally painful or overwhelming to think about cognitive distortions - Daily survey was triggering or overwhelming - Daily cognitive restructuring exercise was repetitive/tedious - Suggestion: randomly present different cognitive distortions or questions to restructure distortions to decrease repetition - Suggestion: add an option to journal within the cognitive restructuring section - Needed better visual design - Exercises were not applicable or relevant to participant - Activity scheduling module was not applicable or relevant to participant - Connection between mood and drinking was not relevant to participant - Relationships module was not relevant to participant - Cognitive restructuring module was not a good fit for participant’s current needs   - Suggestion: add other types of substances to the mood tracker | |
